# Prenatal antioxidant-enriched and pro-oxidant-contained food, IL4 and IL13 pathway genes, and cord blood IgE

**DOI:** 10.1038/s41598-022-06951-9

**Published:** 2022-02-21

**Authors:** Chien-Han Chen, Yungling Leo Lee, Ming-Hsun Wu, Pao-Jen Chen, Tien-Shan Wei, Ching-Ing Tseng, Wei J. Chen

**Affiliations:** 1 Department of Pediatrics, Fu Jen Catholic University Hospital, Fu Jen Catholic University, New Taipei City, Taiwan; 2 School of Medicine, Fu Jen Catholic University, New Taipei City, Taiwan; 3 Department of Pediatrics, Min-Sheng General Hospital, Taoyüan City, Taiwan; 4 Institute of Biomedical Sciences, Academia Sinica, Taipei, Taiwan; 5 Department of Laboratory Medicine, Min-Sheng General Hospital, Taoyüan City, Taiwan; 6 Department of Obstetrics and Gynecology, Min-Sheng General Hospital, Taoyüan City, Taiwan; 7 Centers of Genomic and Precision Medicine, National Taiwan University, Taipei, Taiwan; 8 Institute of Epidemiology and Preventive Medicine, College of Public Health, National Taiwan University, Taipei, Taiwan; 9 Center for Neuropsychiatric Research, National Health Research Institutes, Zhunan, Miaoli County Taiwan

**Keywords:** Applied immunology, Paediatric research, Risk factors, Genetic association study

## Abstract

Prenatal oxidative balance might influence cord blood IgE (cIgE) levels. We aimed to explore if certain prenatal dietary sources of antioxidants and pro-oxidants are associated with cIgE elevation and if they interact with IL4 and IL13 pathway genes. A structured questionnaire was completed during the third trimester of pregnancy for 1107 full-term newborns. Surveyed antioxidant-enriched food included fish, shellfish, and fruit, whereas surveyed pro-oxidant-contained food included fried fish sticks and canned fish. Cord blood was collected for measuring cIgE levels and genotyping *IL13* rs1800925, rs20541, rs848, *IL4* rs2243250, and *STAT6* rs324011. Fairly lean fish consumption showed protection against cIgE elevation (odds ratio [OR] 0.66; 95% CI 0.49–0.90) in the whole sample, while daily fruit (OR 0.46; 95% CI 0.27–0.79) and ≥ monthly canned fish (OR 2.81; 95% CI 1.24–6.36) exhibited associations only in genetically susceptible babies. A prenatal food protective index, comprising any fairly lean fish, daily fruit, and the absence of any canned fish, exerted dose–response protection against cIgE elevation in babies carrying the *IL13* rs20541 GA or AA genotype (*P* for trend < 0.0001; *P* for interaction = 0.004). We concluded that prenatal antioxidant-enriched and pro-oxidant-contained food consumption may influence cIgE, especially in genetically susceptible babies.

## Introduction

Oxidative stress is known to participate in the pathogenesis of allergic sensitization^1^ and asthma^2^, while antioxidants have been shown to suppress immunoglobulin E (IgE) production^3^ and may improve asthma control^4^. In addition, as a part of the pathogenesis, it has been recognized that allergies begin in utero^5^*.* Specific innate and adaptive immune responses that are different from those in healthy individuals may develop in utero, present at birth, and subsequently lead to allergies later in life. Prenatal environments, along with allergy-specific genetic variants, comprise these immunologic and phenotypic differences^5^.

Among prenatal environments, certain protective dietary nutrients (including antioxidants) and potential sources of oxidative stress (such as air pollutant exposures) have been shown to change the risk of childhood/adolescent allergy^6, 7^. Among prenatal foods, fish, known as potential sources of antioxidants, are commonly studied^5, 8, 9^. However, the results to date are contradictory. Some have found fish to protect against, whereas others found it to predispose to, childhood allergic diseases^5, 9–11^. Meanwhile, processed fish, such as fried fish sticks^12^ and canned fish^13^, have been demonstrated to contain pro-oxidants but rarely studied in the prenatal period. Whether the intake of various types of fish, including potential sources of antioxidants or oxidants, alters cord blood total IgE (cIgE) levels, a well-known predictor of childhood allergy^14^, remains unclear.

Furthermore, genetic predisposition is known to be involved in the development of allergies. For example, the single nucleotide polymorphisms (SNPs) of two important T helper 2 (Th2) cytokines (interleukin 4 (IL4) and interleukin 13 (IL13))^15, 16^ and the gene–gene interactions between *IL13* and redox genes^16^ have been associated with elevated cIgE levels. In addition, the results of Gene and Environment Interaction Birth Cohort Study (GEIBCS) in Taiwan revealed gene-environment interactions involving IL4 and IL13 (IL4-IL13) pathway genes and prenatal inhaled environmental exposures to enhance cIgE production^17, 18^. However, whether prenatal food interact with genes to influent cIgE levels remains unknown.

In this study, we used data from the GEIBCS^17, 18^ and hypothesized that certain specific prenatal foods, which were regarded as potential sources of antioxidants or pro-oxidants, would be protectively or adversely associated with cIgE elevation, respectively, and that these associations would be modified by genetic polymorphisms of IL4-IL13 pathway genes.

## Results

### Distributions of prenatal surveyed antioxidant-enriched and pro-oxidant-contained food consumption

The distributions of prenatal consumption of surveyed food with antioxidants and those with pro-oxidants by frequency are shown in Table 1. The proportions of any prenatal intake (more than none) of fairly lean fish, lean fish, moderately fatty fish, fatty fish, shellfish, fried fish sticks, and canned fish were 65.6%, 91.2%, 16.8%, 92.7%, 73.4%, 55.9%, and 54.4%, respectively. The proportions of none, < monthly, and monthly prenatal intake of fruit were extremely low (0.4%, 0.5%, and 1.7%, respectively). Therefore, we combined newborns with these frequencies with those with weekly frequency (32.3%) and considered them the reference group of fruit. Daily fruit (65.1%) would be analyzed as an independent variable in further analysis.

**Table 1:** Distributions of prenatal surveyed antioxidant-enriched and pro-oxidant-contained food consumption by frequencies among term newborns in the Gene and Environment Interaction Birth Cohort Study in Northern Taiwan.

Prenatal food intakes	Frequencies				
	None	< Monthly	Monthly	Weekly	Daily
**Surveyed antioxidant-enriched food**					
Fairly lean fish	381 (34.4)	180 (16.3)	99 (8.9)	444 (40.1)	3 (0.3)
Lean fish	97 (8.8)	168 (15.2)	229 (20.7)	606 (54.7)	7 (0.6)
Moderately fatty fish	921 (83.2)	128 (11.6)	43 (3.9)	15 (1.4)	0 (0.0)
Fatty fish	81 (7.3)	150 (13.6)	208 (18.8)	664 (60.0)	4 (0.4)
Shellfish	294 (26.6)	588 (53.1)	189 (17.1)	36 (3.3)	0 (0.0)
Fruit	4 (0.4)	6 (0.5)	19 (1.7)	357 (32.3)	721 (65.1)
**Surveyed pro-oxidant-contained food**					
Fried fish stick	488 (44.1)	501 (45.3)	81 (7.3)	36 (3.3)	1 (0.1)
Canned fish	505 (45.6)	462 (41.7)	78 (7.1)	60 (5.4)	2 (0.2)

Values are presented as n (%).

### Relations of prenatal surveyed antioxidant-enriched and pro-oxidant-contained food consumption to cIgE elevation

The distributions of prenatal surveyed antioxidant-enriched and pro-oxidant-contained food consumption by and their associations with cIgE elevation are shown in Table 2. Newborns with elevated cIgE had significantly smaller proportions of any fairly lean fish and daily fruit than newborns with normal cIgE (*P* = 0.01 and 0.039, respectively). By univariate logistic regression analysis, any fairly lean fish significantly reduced the risk of cIgE elevation (*P* = 0.007; *P* after FDR = 0.04). Fairly lean fish also exhibited a dose–response protective association (*P* for trend = 0.002). After adjustment for covariates, any fairly lean fish was still protectively associated with cIgE elevation (*P* = 0.008; *P* after FDR = 0.0498) and exerted a dose–response association as well (*P* for trend = 0.002).

**Table 2:** Distributions of prenatal surveyed antioxidant-enriched and pro-oxidant-contained food consumption by and their associations with cord blood total IgE (cIgE) elevation in the study sample (N = 1107).

Prenatal food intakes	cIgE levels^#^		*P*^†^	Group comparison	
	Normal (n = 881)	Elevated (n = 226)		OR (95% CI)^b^	aOR (95% CI)^a^
**Surveyed antioxidant-enriched food**					
Fairly lean fish, any	595 (67.5)	131 (58.0)	0.01	0.66 (0.49–0.89)**^†^	0.66 (0.49–0.90)**^†^
Frequency of fairly lean fish			0.02		
< Monthly	142 (16.1)	38 (16.8)		0.81 (0.53–1.23)	0.80 (0.52–1.22)
Monthly	78 (8.9)	21 (9.3)		0.81 (0.48 (1.38)	0.84 (0.49–1.45)
≥ Weekly	375 (42.6)	72 (31.9)		0.58 (0.41–0.81)**^‡^	0.57 (0.40–0.81)**^‡^
* P* for trend				0.002	0.002
Lean fish, any	809 (91.8)	201 (88.9)	0.17	0.72 (0.44–1.16)	0.69 (0.42–1.12)
Moderately fatty fish, any	153 (17.4)	33 (14.6)	0.32	0.81 (0.54–1.22)	0.81 (0.54–1.23)
Fatty fish, any	819 (93.0)	207 (91.6)	0.48	0.83 (0.48–1.41)	0.78 (0.45–1.35)
Shellfish, any	653 (74.1)	160 (70.8)	0.31	0.85 (0.61–1.17)	0.82 (0.59–1.13)
Fruit, daily	587 (66.6)	134 (59.3)	0.039	0.73 (0.54–0.99)*	0.78 (0.57–1.07)
**Surveyed pro-oxidant-contained food**					
Fried fish stick, any	495 (56.2)	124 (54.9)	0.72	0.95 (0.71–1.27)	0.95 (0.70–1.27)
Canned fish, any	472 (53.6)	130 (57.5)	0.29	1.17 (0.87–1.58)	1.17 (0.87–1.58)

cIgE, cord blood total IgE; OR, odds ratio; aOR, adjusted odds ratio; 95% CI, 95% confidence interval.

^#^Values are presented as n (%).

^†^Chi-square test.

*P < 0.05, **P < 0.01, ^†^*P* < 0.05 after FDR correction, ^‡^*P* < 0.01 after FDR correction.

^a^Adjusted for neonatal sex, gestational age, birth body weight, maternal age, parental education, family history of atopic diseases, environmental tobacco smoke, and perception of mildewy odour.

### Differential associations of fairly lean fish with cIgE elevation by the intake of canned fish

To clarify if there was any association between surveyed antioxidant-enriched food and surveyed pro-oxidant-contained food, we investigated dietary habits. Prenatal intake of any canned fish was associated with more frequent prenatal intake of any fairly lean fish (see Supplementary Table S1 online). Further, any prenatal fairly lean fish was protectively associated with cIgE elevation (OR, 0.63 [95% CI 0.40–0.98]) only in participants without any canned fish. This association disappeared in the presence of any canned fish (see Supplementary Table S2 online).

### No evidence for sex-food interaction

We also tested the models for neonatal sex-by-prenatal food interactions. Prenatal foods were distributed evenly between female and male babies, and there was no significant interaction between sex and prenatal food for cIgE elevation (*P* for interactions were all > 0.05) (see Supplementary Tables S3 and S4 online).

### Gene-food interactions

To explore gene-food interactions, we stratified the total study sample by genetic polymorphisms of IL4-IL13 pathway genes (Table 3) and revealed that daily prenatal fruit intake significantly reduced the risk of cIgE elevation in term babies carrying the *IL4* rs2243250 TC or CC genotype (*P* = 0.005; *P* after FDR = 0.039) but not in those carrying the *IL4* rs2243250 TT genotype, with a significant interaction between daily fruit intake and *IL4* rs2243250 (*P* for interaction = 0.02). Moreover, we found borderline significant interactions between any canned fish and *IL13* rs1800925 and between any canned fish and *IL13* rs20541 (*P* for interaction = 0.049 and 0.0497, respectively). To clarify these relationships, we further examined the canned fish of different frequencies (Table 4), and demonstrated that canned fish significantly enhanced the risk of cIgE elevation in a dose-dependent manner for those carrying the *IL13* rs1800925 CT or TT genotype (*P* for trend = 0.01), with a significant interaction between canned fish and *IL13* rs1800925 (*P* for interaction = 0.02), but not for those carrying *IL13* rs20541 GA or AA genotype.

**Table 3:** Associations of prenatal surveyed antioxidant-enriched and pro-oxidant-contained food consumption with cord blood total IgE elevation modified by genetic polymorphisms.

Prenatal food intake^a^	*IL13* rs1800925		*P*^*#*^	*IL13* rs20541		*P*^*#*^	*IL13* rs848		*P*^*#*^
	CC (n = 797) aOR (95% CI)	CT or TT (n = 310) aOR (95% CI)		GG (n = 496) aOR (95% CI)	GA or AA (n = 611) aOR (95% CI)		CC (n = 501) aOR (95% CI)	CA or AA (n = 605) aOR (95% CI)	
**Surveyed antioxidant-enriched food**									
Fairly lean fish, any	0.70 (0.49–1.02)	0.59 (0.33–1.03)	0.66	0.82 (0.50–1.33)	0.59 (0.39–0.88)**	0.33	0.86 (0.53–1.40)	0.57 (0.38–0.85)**	0.23
Lean fish, any	0.63 (0.36–1.11)	0.87 (0.33–2.33)	0.54	0.68 (0.33–1.42)	0.71 (0.37–1.38)	0.92	1.01 (0.45–2.29)	0.55 (0.29–1.02)	0.25
Moderately fatty fish, any	0.95 (0.59–1.53)	0.53 (0.23–1.26)	0.20	0.61 (0.30–1.21)	1.02 (0.60–1.74)	0.19	0.71 (0.36–1.38)	0.91 (0.53–1.55)	0.53
Fatty fish, any	0.80 (0.41–1.54)	0.73 (0.27–1.99)	0.96	0.69 (0.28–1.70)	0.92 (0.46–1.86)	0.58	1.27 (0.42–3.81)	0.71 (0.37–1.37)	0.41
Shellfish, any	0.76 (0.52–1.13)	0.91 (0.50–1.67)	0.57	0.59 (0.36–0.95)*	1.09 (0.69–1.72)	0.053	0.71 (0.43–1.17)	0.90 (0.58–1.40)	0.43
Fruit, daily	0.78 (0.54–1.13)	0.85 (0.48–1.52)	0.77	1.12 (0.67–1.87)	0.61 (0.41–0.91)*	0.06	1.03 (0.62–1.72)	0.65 (0.44–0.97)*	0.18
**Surveyed pro-oxidant-contained food**									
Fried fish stick, any	0.92 (0.64–1.31)	1.04 (0.59–1.81)	0.75	1.18 (0.74–1.89)	0.82 (0.55–1.21)	0.28	1.19 (0.74–1.91)	0.82 (0.56–1.21)	0.27
Canned fish, any	0.95 (0.66–1.35)	1.86 (1.05–3.30)*	0.049	0.81 (0.51–1.29)	1.53 (1.03–2.28)*	0.0497	0.93 (0.58–1.49)	1.36 (0.92–2.02)	0.25

Prenatal food intake^a^	*STAT6* rs324011		*P*^*#*^	*IL4* rs2243250		*P*^*#*^			
	CC (n = 628) aOR (95% CI)	CT or TT (n = 479) aOR (95% CI)		TT (n = 724) aOR (95% CI)	TC or CC (n = 381) aOR (95% CI)				
**Surveyed antioxidant-enriched food**									
Fairly lean fish, any	0.65 (0.44–0.97)*	0.66 (0.41–1.08)	0.96	0.64 (0.44–0.93)*	0.66 (0.38–1.15)	0.81			
Lean fish, any	0.78 (0.39–1.58)	0.56 (0.28–1.12)	0.51	0.61 (0.35–1.08)	0.95 (0.34–2.65)	0.47			
Moderately fatty fish, any	0.92 (0.54–1.55)	0.68 (0.34–1.36)	0.53	0.84 (0.51–1.39)	0.75 (0.36–1.58)	0.78			
Fatty fish, any	0.57 (0.28–1.16)	1.13 (0.47–2.71)	0.29	0.57 (0.31–1.04)	2.38 (0.54–10.54)	0.04			
Shellfish, any	0.96 (0.61–1.49)	0.63 (0.38–1.04)	0.23	0.84 (0.56–1.26)	0.70 (0.39–1.25)	0.63			
Fruit, daily	0.76 (0.51–1.13)	0.81 (0.49–1.33)	0.76	1.01 (0.68–1.51)	0.46 (0.27–0.79)**^†^	0.02			
**Surveyed pro-oxidant-contained food**									
Fried fish stick, any	0.94 (0.64–1.39)	0.96 (0.60–1.55)	0.94	1.02 (0.70–1.47)	0.77 (0.45–1.30)	0.37			
Canned fish, any	1.25 (0.85–1.84)	1.13 (0.70–1.83)	0.69	1.27 (0.88–1.83)	0.96 (0.57–1.62)	0.37			

aOR, adjusted odds ratio; 95% CI, 95% confidence interval.

^#^*P* for interaction.

*P < 0.05, **P < 0.01, ^†^*P* < 0.05 after FDR correction.

^a^Adjusted for neonatal sex, gestational age, birth body weight, maternal age, parental education, family history of atopic diseases, environmental tobacco smoke, and perception of mildewy odour.

**Table 4:** The dose–response associations of prenatal canned fish with cord blood total IgE elevation modified by *IL13* genetic polymorphisms.

Canned fish^a^	*IL13* rs1800925		*IL13* rs20541	
	CC (n = 797) aOR (95% CI)	CT or TT (n = 310) aOR (95% CI)	GG (n = 496) aOR (95% CI)	GA or AA (n = 611) aOR (95% CI)
None	1	1	1	1
< monthly	0.98 (0.67–1.44)	1.62 (0.87–2.99)	0.75 (0.45–1.24)	1.54 (1.01–2.35)*
≥ monthly	0.84 (0.47–1.49)	2.81 (1.24–6.36)*^†^	1.02 (0.50–2.06)	1.49 (0.81–2.76)
*P* for trend	0.61	0.01	0.66	0.07
*P* for interaction		0.02		0.15

aOR, adjusted odds ratio; 95% CI, 95% confidence interval.

*P < .05, ^†^*P* < .05 after FDR correction.

^a^Adjusted for neonatal sex, gestational age, birth body weight, maternal age, parental education, family history of atopic diseases, environmental tobacco smoke, and perception of mildewy odor.

### Prenatal food protective index (PFPI)

**Figure 1:**
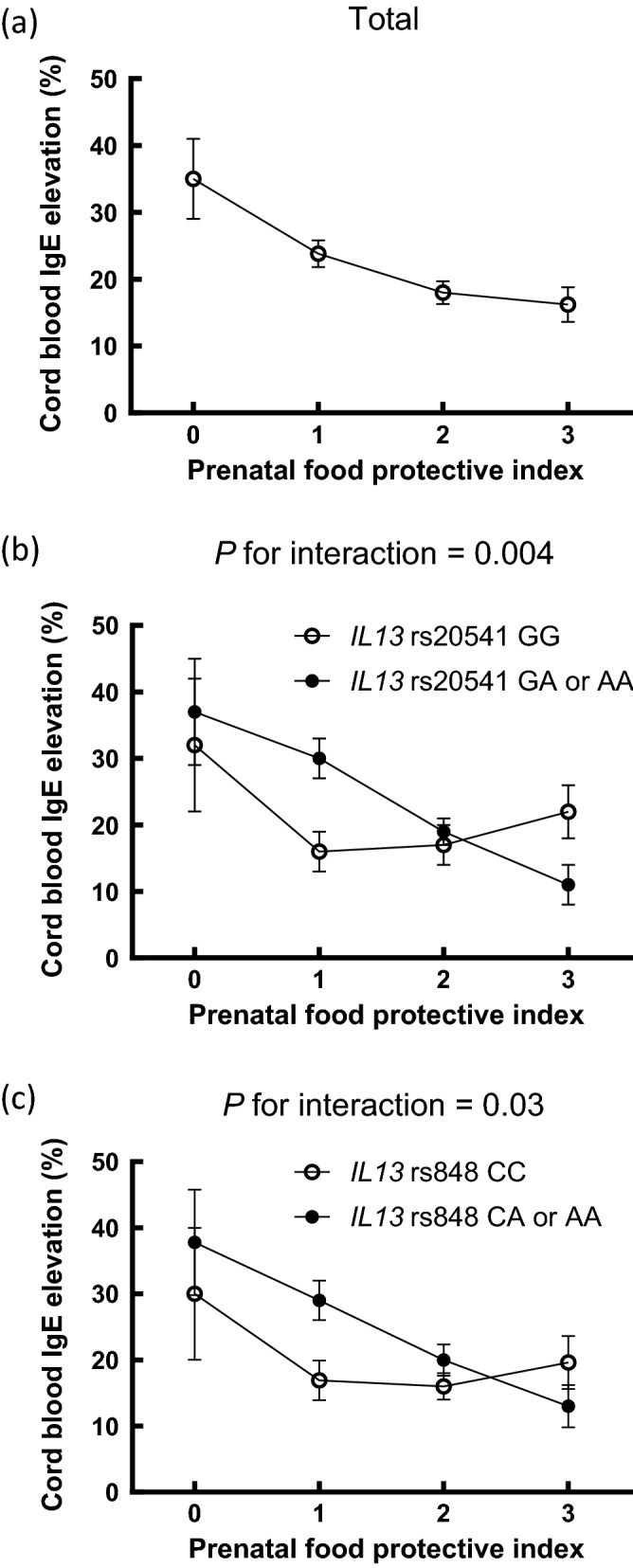
Relations between prenatal food protective index (i.e., any fairly lean fish, daily fruit, and absence of any canned fish) and cord blood total IgE elevation in (**a**) total study sample, (**b**) newborns with *IL13* rs20541 GG genotype or GA or AA genotype, and (**c**) newborns with *IL13* rs848 CC genotype or CA or AA genotype. Data represent proportions ± standard error of the proportion. *P* for interactions are the *P*-values between the prenatal food protective index and *IL13* rs20541 (**b**) and between the prenatal food protective index and *IL13* rs848 (**c**).

To explore whether there is a potentially beneficial dietary pattern, we compiled prenatal intake of any fairly lean fish, daily fruit, and absence of any canned fish into a composite index, namely the prenatal food protective index (PFPI), and participants were divided into four subgroups (i.e., none, one, two, or three protective factors). The corresponding proportion in each food loading category was 60 (5.4%) for none, 340 (30.7%) for one, 509 (46.0%) for two, and 198 (17.9%) for three. We observed a significant dose–response beneficial association of the PFPI with reduced risk for cIgE elevation in the whole study sample (*P* for trend = 0.002) (Table 5). After stratification by genetic polymorphisms, PFPI significantly reduced the risk of cIgE elevation in a dose–response manner in term babies carrying the *IL13* rs20541 GA or AA genotype (*P* for trend =  < 0.0001) or in those carrying the *IL13* rs848 CA or AA genotype (*P* for trend = 0.0002), with significant gene-food matrix interactions (*P* for interaction = 0.004 and 0.03, respectively) (Table 5). Among them, having all three PFPI was associated with 77% (GA or AA of *IL13* rs20541) and 73% (CA or AA of *IL13* rs848) reductions in the risks of cIgE elevation. These interactions are also illustrated as proportions in Fig. 1. For babies having all three PFPI, the proportion ± standard error of cIgE elevation among those carrying *IL13* rs20541 GA or AA genotype (11 ± 3%) was significantly lower than that (22 ± 4%) of babies carrying *IL13* rs20541 GG genotype (*P* = 0.04; *P* for interaction = 0.004).

**Table 5:** Dose–response associations of prenatal food protective index with cord blood total IgE elevation modified by genetic polymorphisms.

PFPI^#,a^	Total	*IL13* rs1800925		*IL13* rs20541		*IL13* rs848	
	(n = 1107) aOR (95% CI)	CC (n = 797) aOR (95% CI)	CT or TT (n = 310) aOR (95% CI)	GG (n = 496) aOR (95% CI)	GA or AA (n = 611) aOR (95% CI)	CC (n = 501) aOR (95% CI)	CA or AA (n = 605) aOR (95% CI)
None	1	1	1	1	1	1	1
1	0.58 (0.32–1.05)	0.71 (0.33–1.54)	0.40 (0.15–1.09)	0.42 (0.15–1.16)	0.77 (0.36–1.62)	0.47 (0.17–1.29)	0.71 (0.34–1.52)
2	0.43 (0.24–0.78)**^‡^	0.55 (0.26–1.19)	0.31 (0.11–0.82)*^†^	0.46 (0.17–1.22)	0.44 (0.21–0.92)*^†^	0.47 (0.18–1.26)	0.44 (0.21–0.92)*^†^
3	0.37 (0.19–0.73)**^‡^	0.53 (0.23–1.24)	0.21 (0.07–0.66)**^†^	0.67 (0.23–1.94)	0.23 (0.09–0.57)**^‡^	0.63 (0.22–1.82)	0.27 (0.11–0.65)**^†^
*P* for trend	0.002	0.09	0.009	0.73	< 0.0001	0.91	0.0002
*P* for interaction			0.15		0.004		0.03

PFPI^#,a^	*STAT6* rs324011		*IL4* rs2243250				
	CC (n = 628) aOR (95% CI)	CT or TT (n = 479) aOR (95% CI)	TT (n = 724) aOR (95% CI)	TC or CC (n = 381) aOR (95% CI)			
None	1	1	1	1			
1	0.52 (0.24–1.15)	0.66 (0.26–1.69)	0.56 (0.26–1.20)	0.53 (0.20–1.40)			
2	0.39 (0.18–0.85)*^†^	0.48 (0.19–1.21)	0.42 (0.20–0.88)*	0.38 (0.14–1.01)			
3	0.32 (0.13–0.75)**^†^	0.43 (0.15–1.27)	0.41 (0.18–0.95)*	0.28 (0.09–0.87)*			
*P* for trend	0.007	0.07	0.03	0.02			
*P* for interaction		0.77		0.62			

PFPI, prenatal food protective index; aOR, adjusted odds ratio; 95% CI, 95% confidence interval.

^#^PFPI includes any fairly lean fish, daily fruit, and absence of any canned fish.

^a^Adjusted for neonatal sex, gestational age, birth body weight, maternal age, parental education, family history of atopic diseases, environmental tobacco smoke, and perception of mildewy odour.

*P < 0.05, **P < 0.01, ^†^*P* < .05 after FDR correction, ^‡^*P* < .01 after FDR correction.

## Discussion

In this study, we evaluated certain prenatal foods with antioxidants or pro-oxidants and demonstrated their associations with cIgE elevation and their interactions with IL4-IL13 pathway genes. Prenatal intake of fairly lean fish was the only food associated with reduced risk for cIgE elevation in the whole study sample. Regarding foods with significant gene-food interactions, prenatal intake of daily fruit in term babies carrying the risk alleles of *IL4* rs2243250 reduced the risk of cIgE elevation. Prenatal intake of canned fish enhanced the risk of cIgE elevation in a dose–response manner in term babies carrying the risk allele of *IL13* rs1800925. Moreover, a summary score PFPI exerted beneficial dose–response associations with reduced risk for cIgE elevation not only in the whole study sample but also in the strata of babies carrying the risk allele of *IL13* rs20541 or *IL13* rs848, with significant gene-food interactions. Among these strata, the potentially beneficial prenatal dietary pattern considering all three indexes resulted in a > 70% reduction in risks.

cIgE elevation increases the risks of allergic diseases in childhood^14^. Despite plenty of studies on the relations between prenatal food and childhood allergies^9^, few focused on cIgE as an early outcome. We demonstrated a reduced risk of cIgE elevation by prenatal intake of fairly lean fish, e.g., flounder and shark, in a dose–response manner in the whole study sample. Flounder is commonly found in the coastal waters of East Asian countries^19^. Flounder muscle contains peptides with high antioxidant activity^20^, and is considered as one of the best choices during pregnancy due to its low mercury level^21^. Moreover, flounder possesses antioxidant defensive responses to mercury. A recent study shows that activities of antioxidants in the flounder muscle are enhanced by mercury, even if its exposure dose is low^22^. Shark muscle is also a natural source of antioxidants^23^. However, because of its high mercury level, the Dietary Guideline of Taiwan cautions pregnant women against eating more than 35 g of shark per week^24^, and the Dietary Guideline for Americans recommends avoidance of shark during pregnancy^21^.

Fatty fish are a rich source of antioxidants too^8^. However, our study did not demonstrate any association between prenatal fatty fish and cIgE elevation, which is consistent with a prenatal fatty fish intervention study^25^. Similarly, there are conflicting reports about its associations with childhood allergic diseases^9–11^. This might be due to the counteracting effect of histamine^26^. Fatty fish is rich in histidine, which is easily transformed to histamine when not adequately cryopreserved^27^.

Prenatal fruit intake has been associated with decreased risk of atopic dermatitis and asthma^9^, while its influence on cIgE remains largely unexplored. Fruits are an important source of dietary antioxidants^28^, including folic acid, vitamin C, and vitamin E, prenatal supplementation of which appear to decrease the risks of atopic dermatitis^29^, allergic sensitization^9^, and infantile wheezing or eczema^9^, respectively. Vitamin E has also been shown to suppress IL-4 secretion in human peripheral blood T cells in a dose-dependent manner^30^, supporting our result of a significant interaction between prenatal fruit intake and *IL4* rs2243250 to reduce cIgE levels.

The influences of prenatal canned fish on cord blood biomarkers remain largely uninvestigated. Canned fish is high in histamine^13^. Histamine not only triggers oxidative stress^31^ but also promote chemotaxis of eosinophils and neutrophils^26^. In addition, histamine has been shown to enhance the production of Th2 cytokines, such as IL-13^32^, and results in elevated serum IgE levels^26^, supporting our finding on the significant interactions between prenatal intake of canned fish and *IL13* rs1800925 in a dose–response manner to increase cIgE levels.

The most ideal prenatal dietary pattern to protect fetuses against cIgE elevation has not been elucidated. Fairly lean fish and fruit are rich sources of antioxidants. One study showed a beneficial combined effect of high prenatal consumption of fish plus fruit^8^, but their influences on cIgE remain unexplored. Antioxidant vitamin C, which is rich in fruit, has a unique ability to catalyze the degradation of histamine^33^, which is rich in canned fish^13^. Our study further revealed that the benefit of fairly lean fish might be counteracted by canned fish. Because of these potential combined effects and mutually counteracted influences, we investigated and established the summary score PFPI, and revealed its dose–response protection against cIgE elevation not only in the whole study sample but also in term babies carrying the risk alleles of *IL13* rs20541 or *IL13* rs848, with significant gene-food matrix interactions. The potentially most beneficial prenatal dietary pattern demonstrated in this study was that including all three indexes.

This study has some limitations. We did not use semi-quantitative food frequency questionnaires^6^ to collect information on prenatal nutrient intake, such as vitamin C, vitamin E, and folic acid. Instead, data on real foods were collected and analyzed, enabling us to suggest a potentially beneficial dietary combination. Besides, we did not include foods extensively. Instead, we incorporated several foods only, based on our hypotheses. Since we did not collect information on maternal body size, maternal asthma history, and maternal asthma status (exacerbated or stable) during pregnancy, we were unable to investigate if these maternal characteristics modified the associations of prenatal foods with cIgE elevation and their interactions with other factors (such as sex and genes). Furthermore, we did not measure prenatal biomarkers of oxidant or antioxidant system, which, based on our exploratory results, warrant further investigation to evaluate their relations to cIgE.

In conclusion, we revealed possibly beneficial (fairly lean fish and fruit) and unfavorable (canned fish) prenatal food for cIgE levels either in the total study sample or in term babies carrying the risk alleles of certain IL4-IL13 pathway genes, with significant gene-food interactions. The potentially most advantageous dietary pattern in this study, which included all three indexes, decreased the risk of cIgE elevation not only in the total study sample but also in the genetic strata, with the largest effect (77% reduction in risk) in term babies carrying the risk allele of *IL13* rs20541. Implementation of this prenatal dietary pattern may be helpful for early prevention of allergies.

## Methods

### The GEIBCS cohort

GEIBCS is a birth cohort study of 1107 Han Chinese term newborns enrolled from March 2008 to April 2011 in Taiwan. Its study design and baseline characteristics have been described in detail elsewhere^17, 18^. Briefly, gestational age was first estimated by maternal report of the first day of the last menstrual period and then determined by a series of periodic gestational ultrasonography. Structured questionnaires^18^ were completed during the third trimester of pregnancy by pregnant women who underwent prenatal care at Min-Sheng General Hospital, and their fetuses were prospectively followed. Full-term birth was defined as the delivery of a baby at or after 37 weeks' gestation. Written informed consent was obtained from the mothers of all participants. This study was approved by the Institutional Review Board of Min-Sheng General Hospital (approval number: MSEIRB0980109). All methods were performed in accordance with the relevant guidelines and regulations.

Among the 1178 newborns recruited, 71 (6%) were excluded due to prematurity (n = 30), respiratory distress (n = 31), Apgar score < 7 at 5 min after birth (n = 7), tetralogy of Fallot (n = 1), severe subgaleal hemorrhage (n = 1), and severe abdominal distention (n = 1). Finally, 1107 term newborns (94%) of Han Chinese ethnicity were included.

### Prenatal surveyed antioxidant-enriched and pro-oxidant-contained food consumption

We collected data on certain prenatal food intake by using our structured questionnaires during the third trimester of pregnancy, which included questions for selected food, but not for total daily food. Prenatal food of interest was divided into two categories: surveyed antioxidant-enriched food vs. surveyed pro-oxidant-contained food. Surveyed antioxidant-enriched food included fish^8^, shellfish (shrimp or crab), and fruit (no specific types)^28^. To contrast and emphasize the potentially inverse effect of certain processing methods on fish^12^, we included fried fish sticks^12^ and canned fish^13^ (tuna, salmon, or sardines) as surveyed pro-oxidant-contained food. Fish were further divided into four subcategories:^10, 11^.

1. Fairly lean fish, containing < 2% fat, which included flounder and shark;
2. Lean fish, containing 2–25% fat, which included codfish, tilapia, weever, whitebait, yellow croaker, whitefish, and abalone;
3. Moderately fatty fish, containing 25–35% fat, which included carp and bonito; and
4. Fatty fish, containing > 35% fat, which included white pomfret, salmon, eel, Pacific saury, milkfish, mullet, and mackerel.

The frequency of prenatal food intake was grouped into five categories: never, rarely (i.e., less than once a month), monthly, weekly, and daily. More than never was defined as any. The fish intake was not energy-adjusted because we did not attempt to estimate the effects of individual dietary components and there has been considerable debate about which of the strategies of energy adjustment is most appropriate^34^.

PFPI was composed of prenatal intake of any fairly lean fish, daily fruit, and absence of any canned fish and was not energy-adjusted. PFPI was calculated by counting the accomplishment of each component of this index. Babies with any one, any two, and all of these 3 protective factors got one point, two points, and three points of PFPI, respectively.

### Outcome measures

Serum cIgE levels were measured using a CAP FEIA system (Pharmacia, Uppsala, Sweden), and levels ≥ 0.5 IU/mL were defined as elevated^16^.

### DNA collection and genotyping

The rationale for choosing three *IL13* SNPs (rs1800925, rs20541, and rs848), *IL4* rs2243250, and *STAT6* rs324011 was described in our previous studies^17, 18^. Genotyping of the five SNPs was performed using TaqMan probe assays (TaqMan^®^ SNP Genotyping Assays, Applied Biosystems, Foster City, CA, USA) on a 7900 Fast Real-Time PCR System (Applied Biosystems). Genotyping of each sample was automatically attributed using SDS 1.3 software for allelic discrimination.

### Statistical analysis

Statistical analyses were performed with SAS software version 9.4 and the SAS/Genetics module (SAS Institute Inc., Cary, NC, USA). The distributions of prenatal surveyed antioxidant-enriched food and pro-oxidant-contained food by cIgE elevation were analyzed by chi-square test. Further, we performed univariate logistic regression to investigate the associations of these prenatal foods with cIgE elevation. We also adjusted for known predictors of atopy (i.e., gestational age, birth body weight, maternal age, and family history of atopic diseases) and correlates of cIgE elevation identified in our previous study^18^ (i.e., neonatal sex, parental education, environmental tobacco smoke, and mildewy odour). The relations of these covariates to prenatal foods and cIgE were illustrated in a directed acyclic graph (see Supplementary Fig. S1 online). Multiple tests were corrected by means of false discovery rate (FDR). Deviations of genotypes from Hardy–Weinberg equilibrium were assessed using the χ^2^ test. Based on the findings of our previous study^17, 18^, the dominant genetic models were implemented.

To further investigate if genetic polymorphisms modified the influences of prenatal surveyed antioxidant-enriched food and pro-oxidant-contained food on cIgE elevation, firstly we stratified the association analyses by genotypes of each genetic polymorphism to explore whether there was any stratum-specific main effect. Significance levels of the associations in each stratification strategy were corrected for multiple testing by means of FDR. A two-tailed *P* value < 0.05 after FDR correction was considered significant. After that, the significance level of statistical interaction was determined using likelihood ratio tests that compared the model with an interaction term versus the model without it.

### Ethical approval

This study was approved by the Institutional Review Board of Min-Sheng General Hospital (approval number: MSEIRB0980109). Written informed consent was obtained from the mothers of all participants.

## Supplementary Information

Supplementary Information.

## Data Availability

Patient data is available upon request with the corresponding author.
