# Supplementary material for: Prenatal antioxidant-enriched and pro-oxidant-contained food, IL4 and IL13 pathway genes, and cord blood IgE
Source: Sci Rep. 2022 Feb 21;12:2884. doi: 10.1038/s41598-022-06951-9 (PMC8861038; doi:10.1038/s41598-022-06951-9)
Supplement: Supplementary file 1 — Supplementary Information. [file 41598_2022_6951_MOESM1_ESM.doc]

**Supplementary Information**

Prenatal antioxidant-enriched and pro-oxidant-contained food, IL4 and IL13 pathway genes, and cord blood IgE

Chien-Han Chen, Yungling Leo Lee, Ming-Hsun Wu, Pao-Jen Chen, Tien-Shan Wei, Ching-Ing Tseng & Wei J. Chen

Table S1. Distributions of prenatal surveyed antioxidant-enriched and pro-oxidant-contained food consumption by canned fish in the study sample.

Table S2. Associations of fairly lean fish and canned fish with cord blood total IgE elevation.

Table S3. Distributions of prenatal surveyed antioxidant-enriched and pro-oxidant-contained food consumption by neonatal sex in the study sample.

Table S4. Univariate logistic regression analysis of cord blood total IgE elevation on prenatal surveyed antioxidant-enriched and pro-oxidant-contained food consumption and their interactions with sex in the study sample.

Figure S1. Directed acyclic graph.

| **Table S1. Distributions of prenatal surveyed antioxidant-enriched and pro-oxidant-contained food consumption by canned fish in the study sample** | | | | | | | |  |
| --- | --- | --- | --- | --- | --- | --- | --- | --- |
|  |  |  | | Canned fish, any | | |  | |
| Prenatal food intakes | | | No (n = 505) | | Yes (n = 602) | *P*^†^ | |  |
| Surveyed antioxidant-enriched food | | |  | |  |  | |  |
|  | Fairly lean fish, any | | 284 (56.2) | | 442 (73.4) | 0.000 | |  |
|  | Lean fish, any | | 429 (85.0) | | 581 (96.5) | 0.000 | |  |
|  | Moderately fatty fish, any | | 78 (15.5) | | 108 (17.9) | 0.27 | |  |
|  | Fatty fish, any | | 453 (89.7) | | 573 (95.2) | 0.001 | |  |
|  | Shellfish, any | | 349 (69.1) | | 464 (77.1) | 0.003 | |  |
|  | Fruit, daily | | 332 (65.7) | | 389 (64.6) | 0.70 | |  |
| Surveyed pro-oxidant-contained food | | |  | |  |  | |  |
|  | Fried fish stick, any | | 218 (43.2) | | 401 (66.6) | 0.000 | |  |
| Values are presented as n (%). | | | | | | | |  |
| ^†^chi-square test. | | | | | | | |  |

| **Table S2. Associations of fairly lean fish and canned fish with cord blood total IgE elevation** | | | | |
| --- | --- | --- | --- | --- |
|  |  | | Canned fish, any | |
| Fairly lean fish, any |  | No | | Yes |
| No |  | 1 | | 1.26 (0.79-2.02) |
| Yes |  | 0.63 (0.40-0.98)* | | 0.81 (0.54-1.19) |
| The reference group is participants without canned fish and without fairly lean fish. | | | | |
| Values are presented as odds ratio (95% confidence interval). | | | | |
| **P* < .05. | | | | |

| **Table S3. Distributions of prenatal surveyed antioxidant-enriched and pro-oxidant-contained food consumption by neonatal sex in the study sample** | | | | |
| --- | --- | --- | --- | --- |
| Prenatal food intakes | | Female (n = 546) | Male (n = 561) | *P*^†^ |
| Surveyed antioxidant-enriched food | |  |  |  |
|  | Fairly lean fish, any | 362 (66.3) | 364 (64.9) | 0.62 |
|  | Lean fish, any | 496 (90.8) | 514 (91.6) | 0.65 |
|  | Moderately fatty fish, any | 99 (18.1) | 87 (15.5) | 0.24 |
|  | Fatty fish, any | 501 (91.8) | 525 (93.6) | 0.24 |
|  | Shellfish, any | 400 (73.3) | 413 (73.6) | 0.89 |
|  | Fruit, daily | 359 (65.8) | 362 (64.5) | 0.67 |
| Surveyed pro-oxidant-contained food | |  |  |  |
|  | Fried fish stick, any | 312 (57.1) | 307 (54.7) | 0.42 |
|  | Canned fish, any | 290 (53.1) | 312 (55.6) | 0.40 |
| Values are presented as n (%). | | | | |
| ^†^chi-square test. | | | | |

| **Table S4. Univariate logistic regression analysis of cord blood total IgE elevation on prenatal surveyed antioxidant-enriched and pro-oxidant-contained food consumption and their interactions with sex in the study sample** | | | | |
| --- | --- | --- | --- | --- |
| Prenatal food intakes | | Female (n = 546) OR (95% CI) | Male (n = 561) OR (95% CI) | *P* for interaction |
| Surveyed antioxidant-enriched food | |  |  |  |
|  | Fairly lean fish, any | 0.93 (0.58-1.48) | 0.52 (0.35-0.78)** | 0.07 |
|  | Lean fish, any | 0.47 (0.24-0.92)* | 1.04 (0.51-2.11) | 0.11 |
|  | Moderately fatty fish, any | 0.87 (0.48-1.59) | 0.80 (0.46-1.39) | 0.83 |
|  | Fatty fish, any | 0.92 (0.41-2.04) | 0.70 (0.34-1.47) | 0.63 |
|  | Shellfish, any | 0.74 (0.45-1.21) | 0.93 (0.60-1.44) | 0.49 |
|  | Fruit, daily | 0.95 (0.59-1.53) | 0.61 (0.41-0.90)* | 0.15 |
| Surveyed pro-oxidant-contained food | |  |  |  |
|  | Fried fish stick, any | 0.90 (0.57-1.41) | 1.01 (0.68-1.49) | 0.71 |
|  | Canned fish, any | 1.09 (0.70-1.72) | 1.22 (0.82-1.80) | 0.73 |
| OR, odds ratio; 95% CI, 95% confidence interval. | | | | |
| **P* < .05, ***P* < .01. | | | | |

a

b

c

d

Prenatal foods

Cord blood total IgE

e

f

g

a= maternal age

b= parental education

h

c= family history of atopic diseases

d= environmental tobacco smoke

e= mildewy odour

f= neonatal sex

g= gestational age

h= birth body weight

Figure S1. Directed acyclic graph. The relations of eight covariates to the exposure (prenatal foods) and outcome (cord blood total IgE) were illustrated.
